# Malnutrition and Alcohol in Patients Presenting with Severe Complications of Cirrhosis After Laparoscopic Bariatric Surgery

**DOI:** 10.1007/s11695-021-05237-9

**Published:** 2021-01-23

**Authors:** Yuly P. Mendoza, Chiara Becchetti, Tao Wan, Philipp Nett, Susana G. Rodrigues, Jean-François Dufour, Annalisa Berzigotti

**Affiliations:** 1 University Clinic for Visceral Surgery and Medicine, Inselspital, University of Bern, Freiburgstrasse 18, 3010 Bern, Switzerland; 2 Department of Biomedical Research, University of Bern, Bern, Switzerland; 3 Department of Hepatobiliary Surgery, The Third Xiangya Hospital, Central South University, Changsha, Hunan China

**Keywords:** Roux-en-Y-gastric-bypass, Sleeve gastrectomy, Sarcopenia, Alcohol use disorder

## Abstract

Factors and outcomes associated with decompensation of liver disease and liver failure in obese patients who underwent modern bariatric surgery are unclear. We present here a cohort of seventeen consecutive patients referred because of decompensation of liver disease following laparoscopic bariatric surgery. All patients showed signs of malnutrition (sarcopenia in 76.5%). In ten (58.8%), decompensation was associated with alcohol ingestion, which started after bariatric surgery in six patients. One patient died and three patients required liver transplantation, in one case preceded by transjugular intrahepatic portosystemic shunt (TIPS). However, thirteen patients achieved stabilization or full re-compensation with medical therapy and nutritional support. Our cases underline the risk of alcohol intake and malnutrition after laparoscopic bariatric surgery as causes of severe liver decompensation and underline the need for careful interdisciplinary care of these patients after surgery to early identify and treat alcohol misuse, malnutrition, and liver disease.

**Supplementary Information:**

The online version contains supplementary material available at 10.1007/s11695-021-05237-9.

## Introduction

Obesity has grown dramatically in the last 20 years, becoming rapidly a public health issue. This led to a massive increase in cases of non-alcoholic fatty liver disease (NAFLD), or as most recently defined, metabolic associated fatty liver disease (MAFLD) [1], which can progress to end-stage liver disease and development of hepatocellular carcinoma. Lifestyle modifications are the first-line therapy for obesity and NAFLD, but only a minority of patients achieve clinically relevant and durable weight loss. Bariatric surgery is an effective therapeutic approach for morbid obesity and for patients with obesity presenting metabolic complications and not responding to lifestyle changes [2].

Bariatric procedures currently used include restrictive bowel surgery (i.e., laparoscopic adjustable gastric banding and sleeve gastrectomy), and procedures combining malabsorption and restriction (i.e., biliopancreatic diversion (BPD), and Roux-en-Y-gastric-bypass (RYGB) [2]. Pure malabsorptive procedures, such as jejunoileal bypass, have been abandoned because of the high long-term complication rates including liver failure. Rapid weight loss and severe post-operative malnutrition might indeed lead to liver disease progression to cirrhosis and its complications [3–5].

Studies have shown a high prevalence, ranging from 12 to 20% of alcohol use disorder (AUD) after bariatric surgery [6, 7]. However, data describing the features of decompensation of liver disease after laparoscopic bariatric surgery according to alcohol consumption are lacking.

This study aimed at depicting the clinical characteristics of a consecutive cohort of patients who developed liver dysfunction and clinical decompensation of liver disease after currently practiced techniques of bariatric surgery.

## Methods

We retrospectively assessed all patients consecutively observed due to clinical decompensation of liver disease occurring after bariatric surgery at academic center between February 2014 and February 2020. Patients were followed until May 2020.

Pre- and post-operative data collected included anthropometric variables, comorbidities, laboratory, radiology results, nutritional and metabolic complications, and alcohol consumption. The presence and severity of liver disease before surgery was carefully investigated. We recorded the type of bariatric surgery and presence and type of intra- and post-operative complications. In the follow-up, we collected data on the type of clinical decompensation (ascites, portal hypertensive bleeding, or hepatic encephalopathy) and other complications of cirrhosis (e.g., severe bacterial infections). We calculated the following since surgery: (a) weight loss in kilogram; (b) percent total weight loss (%TWL); and (c) percent excess weight loss (%EWL).

Outcomes included recovery, further decompensation, death, or liver transplantation. The study was executed in accordance with the Helsinki declaration. All patients included in the present study signed the general consent allowing reuse of their health-related data. A study-specific informed consent was not required.

## Results

Seventeen patients (64.7% male, median age of 55.2 years [IQR 49–63]) presented clinical decompensation of liver disease after laparoscopic bariatric surgery in the study period. Surgery had been performed in other centers in the majority of cases (*n* = 11, 65%). Preoperative body weight and BMI were 127.0 ± 23.2 kg and 42.9 ± 6.3 kg/m^2^, respectively.

Eleven patients (65%) had received a non-invasive assessment of liver disease before bariatric surgery. Among them, three patients showed no liver disease, three (18%) had NAFLD without severe fibrosis and five patients (29%) had cirrhosis. All patients with cirrhosis were in compensated stage, Child-Pugh A5-B7 and MELD 6–9 points. In the remaining 6 patients, no information regarding a possible pre-existing liver disease was available (they had been seen and operated on in other centers).

Among the five patients diagnosed with cirrhosis by non-invasive tests and clinical findings, one was biopsied before the surgical intervention, and cirrhosis was confirmed. The other four received biopsy during bariatric surgery showing bridging fibrosis and mild steatohepatitis in two, and cirrhosis in two. One additional patient underwent liver biopsy during surgery and histology showed nodular regenerative hyperplasia (NRH).

Laparoscopic RYGB was used in eleven cases (65%), laparoscopic sleeve gastrectomy in five (29%), and laparoscopic gastric banding in one (6%). The latter did not achieve a satisfactory weight loss, and later on, underwent laparoscopic biliopancreatic diversion with duodenal switch procedure (BPD-DS). Three patients (18%) developed surgical complications (anastomotic leak) requiring re-laparoscopy and drainage. No surgical mortality was observed. The intervention led to a significant weight reduction in all, but in two cases. The median BMI, %EWL, and %TWL at the time of clinical decompensation were 25.4 kg/m^2^ (range16.7–36.4 kg/m^2^), 96.8% (35–163%), and 36.6% (13–60%), respectively.

### Features of Clinical Decompensation of Liver Disease

**Fig. 1:**
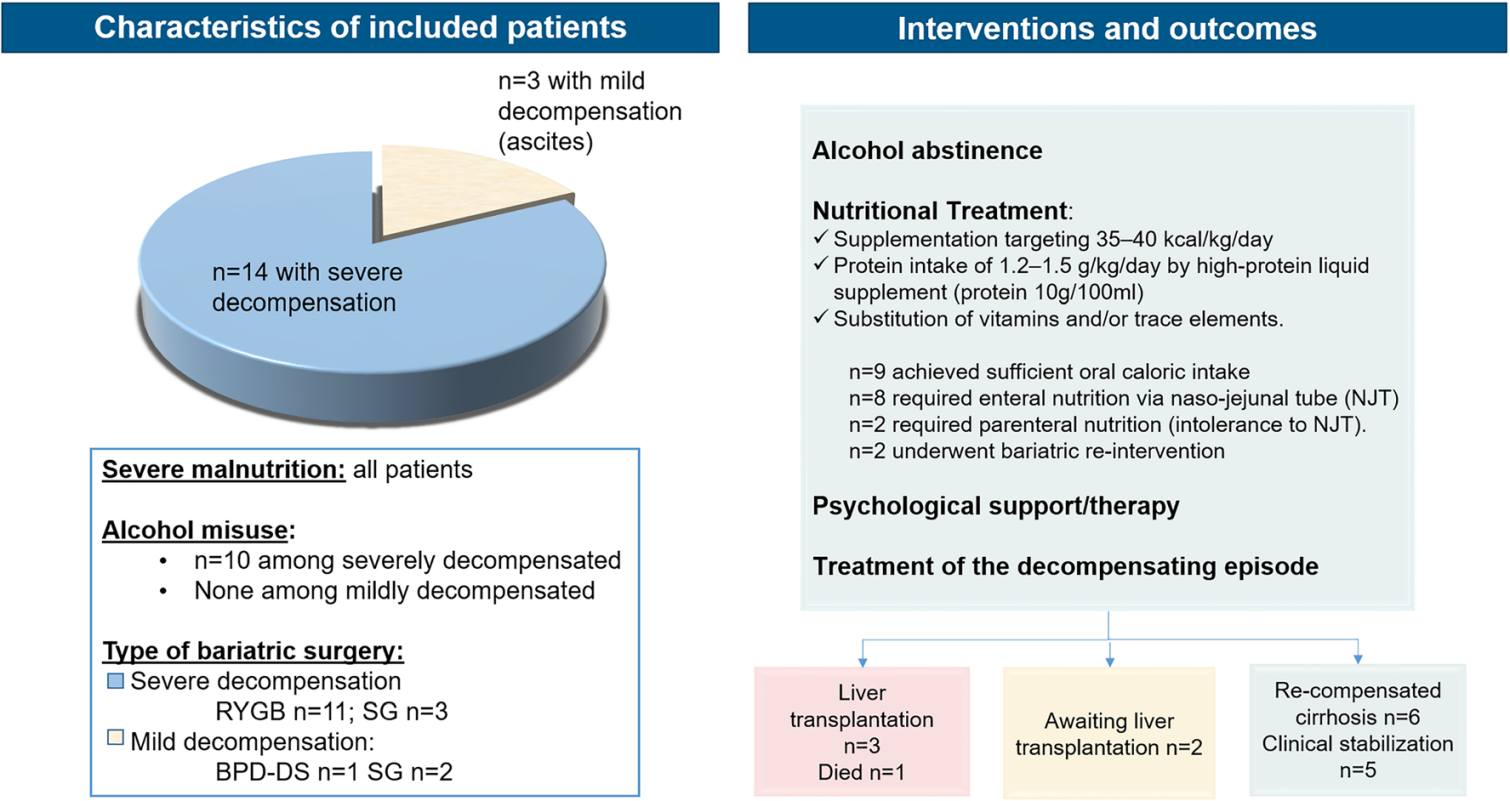
Characteristics of bariatric surgery, clinical course, and clinical decompensation in the study population. RYGB, Roux-en-Y-gastric-bypass; SG, sleeve gastrectomy; BPD-DS, biliopancreatic diversion with duodenal switch procedure; HRS, hepatorenal syndrome; SBP, spontaneous bacterial peritonitis; HE, hepatic encephalopathy; AH, alcoholic hepatitis

Clinical decompensation of liver disease developed in a median of 33 months (IQR 8–73) after surgery. At presentation, decompensation was severe in 14 cases (82%; median MELD score 18 points) and mild in 3 (18%; median MELD score 6 points). The characteristics of bariatric surgery, clinical course, and clinical decompensation in the study population are shown in Fig. 1 and more in detail in Supplementary Table 1.

Ten patients (58.8%) presented excessive alcohol consumption associated with AUD at the time of severe decompensation. Among the 10 cases, three patients reported an alcohol intake before surgery but none of them had a recognized AUD, which they developed after surgery. The remaining six patients started drinking alcohol 1–4 years after bariatric surgery. The amount of alcohol consumption varied between 30 and 400 g/day. As for the cofactors related to alcohol intake, 50% of patients had been diagnosed with depression. Clinical decompensation was severe in all 10 patients with alcohol misuse.

In the two patients with alcoholic hepatitis, the Maddrey discriminant function was 17 and 77. Severe bacterial infections were present in five patients, with three being critically ill, requiring intensive care.

As for the nutritional status, thirteen patients (76.5%) received a diagnosis of sarcopenia, in two cases associated with persistent obesity (sarcopenic obesity). The remaining four (23.5%) were diagnosed with severe malnutrition (cachexia). Ten patients had a complete nutritional assessment at the time of decompensation showing low pre-albumin serum levels 0.06 ± 0.05 g/L, as well low albumin 25.5 ± 6 g/L, zinc 8.69 ± 2.74 μmol/L, magnesium 0.79 ± 0.28 μmol/L, and selenium 0.72 ± 0.26 μmol/L.

Patients with and without alcohol intake had similar weight loss (*P* = 0.823), BMI (*P* = 0.778), %EWL (*P* = 0.849), and %TWL (*P* = 0.761).

All received a nutritional assessment by nutrition specialists, who prescribed oral supplementation targeting 35–40 kcal/kg/day, as well as protein intake of 1.2–1.5 g/kg/day by high-protein liquid supplement (protein 10 g/100 ml), plus substitution of vitamins and/or trace elements. Eight (47%) patients required further enteral nutrition supplemented through nasojejunal tube (NJT) for 1 to 4 months, two (12%) of them had intolerance to NJT and required parenteral nutrition.

Two patients underwent bariatric re-intervention. Laparoscopic reversal of bariatric surgery was performed in the patient who previously underwent BPD-DS. In another patient, who had undergone RYGB, a proximalization of the lower anastomosis was performed 2 years after the first surgery. Both surgical procedures resulted in improvement of malnutrition and clinical stabilization of liver disease.

The duration of the first hospitalization was in median of 20 days (IQR 7–28). All patients were maintained on nutritional supplementation and alcohol abstinence.

At the end of follow-up, eleven (65%) patients were alive without liver transplantation, three (18%) patients underwent liver transplantation (in one case after TIPS for refractory ascites), two patients (12%) are currently on the liver transplantation waiting list in a stable clinical situation and one (6%) died due to end-stage liver disease (sepsis, ascites, and hepatic hydrothorax).

**Fig. 2:**
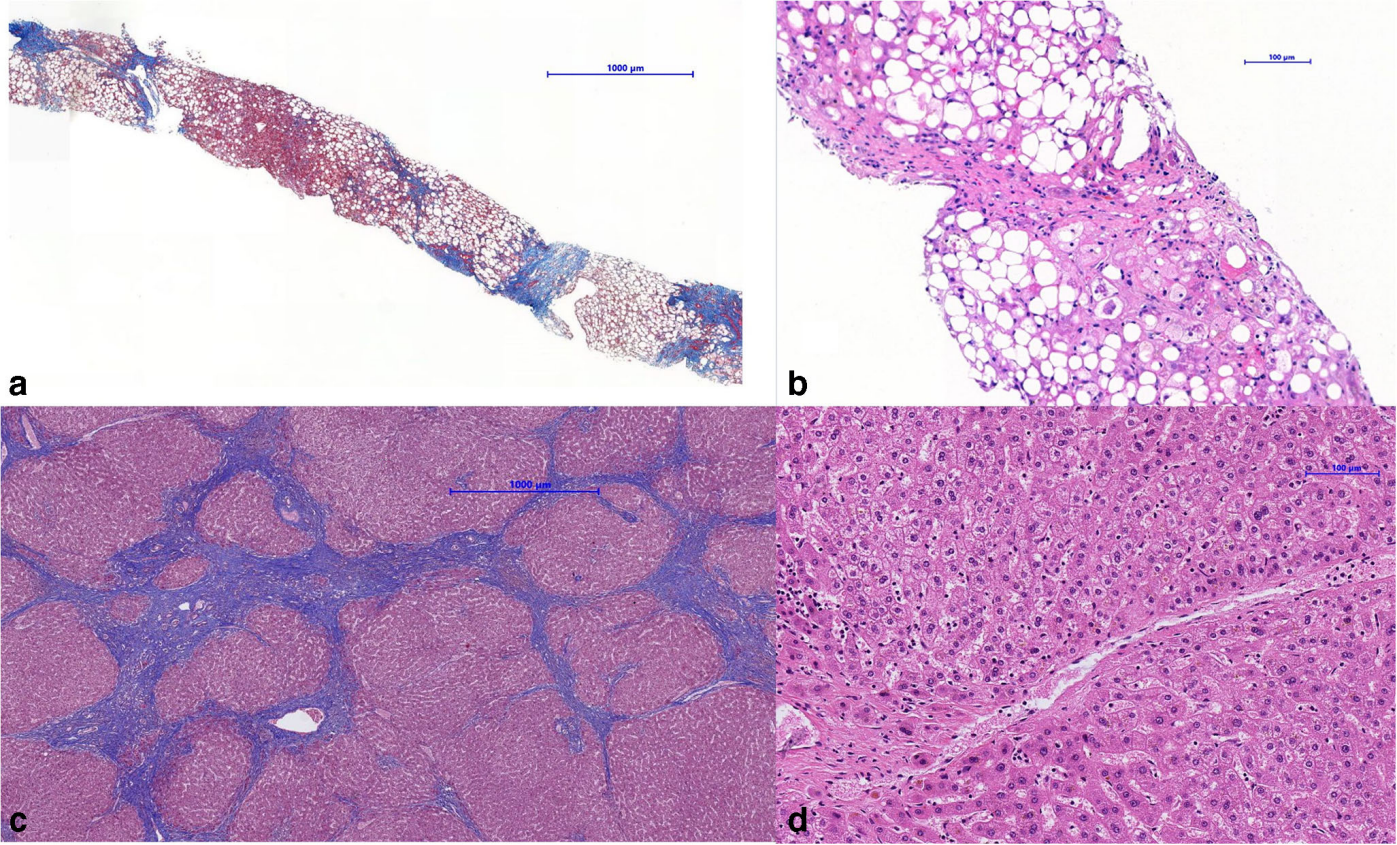
Histology at the time of decompensation showed cirrhosis and severe macrovesicular steatosis, affecting >95% of hepatocytes, ballooning of hepatocytes, and lobular inflammation (panels **a**, **b**). The patient was listed for transplantation. He received enteral nutrition and microelements. His liver function improved (from Child-Pugh 11 to 7 points) during the waiting time. Histology of the explant (panels **c**, **d**) showed cirrhosis without steatosis and mild unspecific porto-septal inflammation

The histological findings of one of the described cases who achieved improvement after enteral nutrition and was successfully transplanted are shown in Fig. 2.

## Discussion

In the present series, we described the features and outcomes of patients presenting liver decompensation after bariatric surgery. Many of them presented with very severe liver dysfunction and complications of portal hypertension, and bacterial infections were common. Almost two-thirds of patients reported excessive alcohol consumption. Notably, some of them started drinking alcohol after bariatric surgery (one to 4 years after).

In our cohort, none of the patients had a recognized AUD prior to surgery, and patients began misusing alcohol after bariatric surgery. There is experimental and clinical evidence suggesting that bariatric surgery increases the risk of alcohol misuse [6, 8]. Among the mechanisms of “de novo” alcohol addiction, it has been suggested that after bariatric surgery patients experience an “addiction transfer” replacing the old addiction (food) with a new one (alcohol). This could explain the pattern of “de novo” AUD in our patients. Neuro-hormonal mechanisms determining changes in reward processing and dopamine signaling are involved [9]. Additionally, hormones as ghrelin and glucagon-like peptide-1 (GLP-1), which change after bariatric surgery and play a role in the gut-liver-brain axis, may modulate the dopamine reward system, leading to an increased sensitivity to rewards or craving following bariatric surgery. In this “primed” system, alcohol easily becomes an alternative reward to food [8, 9]. Another not exclusive hypothesis is that alcohol intoxication can take place more quickly with less alcohol and lasts longer in this situation.

Over one-third (41%) of patients in the present series developed decompensation of liver disease in the absence of alcohol consumption. Although it is well established that bariatric surgery is effective in patients with non-alcoholic steatohepatitis (NASH) and obesity, leading to resolution of NASH in up to 80–100% and to fibrosis improvement [2], some reports described that a drastic and rapid weight loss (over 1.6 kg/week) can worsen the course of liver disease. Rapid weight loss leads in fact to rapid mobilization of extrahepatic fat resulting in increases in free fatty acids and proinflammatory cytokines, with worsening of steatohepatitis lobular activity [3, 10]. Mechanisms leading to steatohepatitis in this clinical scenario are not completely understood. On the one side, the rapid weight loss after bariatric surgery produces fast and massive destruction of adipose tissue in visceral and peripheral deposits, leading to release of free fatty acids (FFA) in plasma, reaching the liver through the portal vein [5]. It has also been hypothesized that after bariatric surgery there could be an alteration in bacterial overgrowth of the excluded small intestine, promoted by absence of bile acids, decreased gastric acidity, and dysmotility, which lead to mucosal injury and increase gut permeability to endotoxins [3].

In our study, five patients presented decompensation of liver disease after sleeve gastrectomy. Importantly, all had a diagnosis of compensated cirrhosis prior to bariatric surgery. Compensated cirrhosis without severe portal hypertension is not considered a contraindication to sleeve gastrectomy [11], which has the advantage of interrupting the short gastric veins, often feeding gastroesophageal varices. However, severe malnutrition, micronutrient deficiencies, and sarcopenia can anyhow occur leading to dramatic deterioration of liver function.

Our findings suggest that strict follow-up of weight loss and supplementation of vitamins and trace elements should be performed even more carefully in patients with known liver disease to avoid further progression in those with pre-cirrhotic stages, and to prevent decompensation in those with cirrhosis. In addition, alcohol consumption should be specifically screened and cessation should be recommended. The set of actions that in our view can be put in place to early detect liver disease, follow-up its course after bariatric surgery, and potentially avoid clinical decompensation are summarized in Table 1.

**Table 1:** Suggested actions to identify and follow-up liver disease and to potentially avoid severe decompensation in patients undergoing bariatric surgery. As shown, this population requires the work of an interdisciplinary team

Aspect to address	Before bariatric surgery	After bariatric surgery	Main responsible
Alcohol misuse	• Screen for alcohol use disorder using for instance Alcohol Use Disorder Identification Test concise (AUDIT-C) • Recommend alcohol cessation	• Screen for alcohol use disorder using for instance AUDIT-C • Reinforce the need for alcohol use cessation • Test for AST, ALT and GGT • If alcohol consumption remains unclear: test for carbohydrate-deficient transferrin (CDT) or urinary ethyl glucuronide (uETG)	• All the physicians involved in the case • Referral to a psychiatrist if AUDIT-C positive • Referral to a hepatologist after surgery whenever alcohol consumption or altered liver tests is detected
Depression	• Screen and treat if needed	• Screen and treat if needed	• Psychologist (screening) • Referral to a psychiatrist if needed
Assess the presence and severity of liver disease	• Non-invasive tools: ○ Laboratory tests: AST, ALT, ALP, albumin, bilirubin, INR, platelet count ○ Ultrasound ○ Liver stiffness measurement (transient elastography; magnetic resonance elastography) • In case of possible relevant liver disease: liver biopsy • In case of suspected cirrhosis: liver biopsy using transjugular route and hepatic venous pressure gradient measurement • If portal hypertension is confirmed, upper GI tract endoscopy to rule out varices	• In patients with abnormal liver tests before surgery or with abnormal results on intraoperative liver biopsy: regular follow-up at the liver clinic using the same tests • Suggested timing after surgery: 3–6 months; 12 months and then every 1–2 years if normal findings at 12 months	• Hepatologist
Sarcopenia and nutrition supplementation	• Baseline measurement of skeletal muscle index/psoas area if computerized tomography is available • Baseline hand-grip strength test • Increase physical activity and decrease sedentary lifestyle.	• Strictly monitor weight loss • Consider serial hand-grip strength tests to follow-up physical performance; single slice CT for muscle mass • Monitor and treat for micronutrient deficiency (i.e., zinc, calcium, vitamin D). If signs of malnutrition (especially in sarcopenic obese or if too quick weight loss): • High-protein intake (aim for 1.5-2 g/Kg/day) • Decrease fasting times—snack/meals every 3–4 h and late evening snack • Reinforce: increase physical activity and decrease sedentary lifestyle.	• Endocrinologist expert in clinical nutrition • Early referral to hepatologist in patients with pre-existing liver disease or alteration of liver tests
Osteoporosis		• Monitor bone mineral density: dual X-ray energy absorptiometry (DEXA) at spine and hip.	• Endocrinologist

In conclusion, in our cohort, which is the largest so far reporting on liver decompensation in patients who underwent modern techniques of bariatric surgery, alcohol use and severe malnutrition triggered severe episodes of decompensation of liver disease. Bacterial infections commonly complicated the course of decompensation, and an appropriate nutritional supplementation led to stabilization and even reversal of the course of liver disease back to a fully compensated status. Early detection of malnutrition, prevention, and early recognition of alcohol use disorder, and identification of other risk factors for liver disease progression and decompensation in this population should be addressed by future research.

## Supplementary Information

ESM 1 (DOCX 18 kb).

## Abbreviations

BMI: Body mass index
ALT: Alanine aminotransferase
AUD: Alcohol use disorder
GGT: Gamma-glutamyl transpeptidase
INR: International normalized ratio
OSA: Obstructive sleep apnea
HVPG: Hepatic venous pressure gradient;
LS: Liver stiffness
BS: Bariatric surgery
LD: Liver dysfunction
%EWL: Percent excess weight loss
%TWL: Percent total weight loss
BPD-DS: Biliopancreatic diversion with duodenal switch procedure
RYGB: Roux-en-Y-gastric-bypass
NRH: Nodular regenerative hyperplasia
HRS: Hepatorenal syndrome
SBP: Spontaneous bacterial peritonitis
HE: Hepatic encephalopathy
AH: Alcoholic hepatitis
NJT: Nasojejunal tube
OH: Alcohol
PH: Portal hypertensive
TIPS: Transjugular Intrahepatic Portosystemic Shunt
OLT: Orthotopic liver transplantation
MELD: Model for End-stage Liver Disease
ALD: Alcohol-related liver disease
AHHS: Alcoholic Hepatitis Histologic Score
