# Supplementary material for: Malnutrition and Alcohol in Patients Presenting with Severe Complications of Cirrhosis After Laparoscopic Bariatric Surgery
Source: Obes Surg. 2021 Jan 23;31(6):2817–22. doi: 10.1007/s11695-021-05237-9 (PMC8113216; doi:10.1007/s11695-021-05237-9)
Supplement: Supplementary file 1 — (DOCX 18 kb). [file 11695_2021_5237_MOESM1_ESM.docx]

**Malnutrition and alcohol in patients presenting with severe complications of cirrhosis after bariatric surgery.**

Supplementary table 1…………………………………………………………………….2

**Supplementary Table 1**. Characteristics of bariatric surgery, clinical course and clinical decompensation in the study population.

| **Age / sex** | **Type of laparoscopic bariatric surgery** | **Weight loss (kg)** | **%TWL** | **%EWL** | **Type of decompensation and presentation** | **Months since surgery** | **Nutritional Treatment** | **Final Outcome** |
| --- | --- | --- | --- | --- | --- | --- | --- | --- |
| 31/F | RYGB | 64 | 60.4 | 135.8 | Severe ascites, then 5 months later variceal bleeding | 45 | Enteral nutrition by NJT | Re-compensated cirrhosis |
| 55/M | RYGB | 42 | 30.7 | 56.4 | Subacute liver failure (ascites, SBP, HE, HRS) | 7 | Enteral nutrition by NJT | Underwent liver transplantation after 6 months |
| 51/M | RYGB | 65 | 36.1 | 62.6 | Jaundice, ascites, HE, HRS and peritonitis (perforation) | 8 | Enteral nutrition by NJT | Re-compensated cirrhosis |
| 54/M | RYGB | 60 | 36.1 | 66.1 | Variceal bleeding (1 months later rebleeding from fundal varices) and ascites. The patient received TIPS. | 96 | Oral supplementation | Re-compensated cirrhosis |
| 56/F | Sleeve  Gastrectomy | 15 | 13.6 | 31.1 | Ascites | 3 | Oral supplementation | Clinical stabilization |
| 44/M | RYGB | 42 | 37.2 | 87.3 | Jaundice, HRS, HE and pneumonia in the context of AH | 39 | Oral supplementation -OH abstinence | Re-compensated cirrhosis |
| 54/M | Sleeve  Gastrectomy | 57 | 50.0 | 138.6 | Variceal bleeding, ascites, then HE 6 months later | 11 | Parenteral nutrition + NJT | Clinical stabilization |
| 45/F | RYGB | 51 | 48.1 | 129.0 | Refractory ascites, then septic shock (abdominal origin) with HE and HRS 2 months later | 48 | Enteral nutrition by NJT | Underwent liver transplantation after 9 months |
| 48/F | RYGB | - | - | . | Ascites, SBP, then recurrent HE | 12 | Surgery with proximalization of the lower anastomosis | Clinical stabilization |
| 41/M | RYGB | 56 | 38.3 | 92.3 | Ascites, HE | 63 | Oral supplementation | Clinical stabilization |
| 58/M | Sleeve  Gastrectomy | 25 | 24.0 | 68.0 | Ascites | 8 | Oral supplementation | Clinical stabilization |
| 34/M | RYGB | 62 | 43.7 | 86.6 | Ascites, SBP and HE | 84 | Oral supplementation | Awaiting transplantation |
| 56/M | RYGB | 40 | 32.3 | 86.7 | Ascites and Hepatic hydrothorax | 9 | Oral supplementation | Re-compensated cirrhosis |
| 60/F | Sleeve  Gastrectomy | 23 | 21.7 | 53.9 | Jaundice in the context of AH | 34 | Enteral nutrition by NJT and OH abstinence | Awaiting transplantation |
| 61/M | Sleeve  Gastrectomy | 43 | 41.7 | 106.4 | Ascites, Hepatic hydrothorax, HE and sepsis unclear focus | 24 | Enteral nutrition by NJT | Died |
| 64/F | BPD-DS |  | 55.9 | 97.6 | Ascites | 120 | Reversal surgery of BPD-DS | Re-compensated cirrhosis |
| 40/M | RYGB | 25 | 17.5 | 37.4 | Refractory ascites, HRS, then 2 months later SBP, HRS-hemodialysis, recurrent abdominal infections and PH bleeding requiring TIPS | 96 | Enteral nutrition by NJT | Underwent liver transplantation after 10 months |

Abbreviations: BS, Bariatric surgery; LD, Liver dysfunction; %EWL, percent excess weight loss; %TWL, percent total weight loss; BPD-DS, biliopancreatic diversion with duodenal switch procedure; RYGB, Roux-en-Y-gastric-bypass; HRS, Hepatorenal syndrome; SBP, Spontaneous bacterial peritonitis; HE, hepatic encephalopathy; AH, Alcoholic hepatitis; NJT, naso-jejunal tubes; OH, alcohol; PH, Portal hypertensive; TIPS, Transjugular Intrahepatic Portosystemic Shunt
